# Association between Fluoxetine Use and Overall Survival among Patients with Cancer Treated with PD-1/L1 Immunotherapy

**DOI:** 10.3390/ph16050640

**Published:** 2023-04-23

**Authors:** Joseph Magagnoli, Siddharth Narendran, Felipe Pereira, Tammy H. Cummings, James W. Hardin, S. Scott Sutton, Jayakrishna Ambati

**Affiliations:** 1 Dorn Research Institute, Columbia VA Health Care System, Columbia, SC 29209, USA; 2 Department of Clinical Pharmacy and Outcomes Sciences, College of Pharmacy, University of South Carolina, Columbia, SC 29208, USA; 3 Center for Advanced Vision Science, University of Virginia School of Medicine, Charlottesville, VA 22903, USA; 4 Department of Ophthalmology, University of Virginia School of Medicine, Charlottesville, VA 22903, USA; 5 Department of Epidemiology & Biostatistics, University of South Carolina, Columbia, SC 29208, USA; 6 Department of Pathology, University of Virginia School of Medicine, Charlottesville, VA 22903, USA; 7 Department of Microbiology, Immunology, and Cancer Biology, University of Virginia School of Medicine, Charlottesville, VA 22903, USA

**Keywords:** checkpoint inhibitors, NLRP3, PD-1/L1 immunotherapy, fluoxetine

## Abstract

Checkpoint inhibitors can be a highly effective antitumor therapy but only to a subset of patients, presumably due to immunotherapy resistance. Fluoxetine was recently revealed to inhibit the NLRP3 inflammasome, and NLRP3 inhibition could serve as a target for immunotherapy resistance. Therefore, we evaluated the overall survival (OS) in patients with cancer receiving checkpoint inhibitors combined with fluoxetine. A cohort study was conducted among patients diagnosed with lung, throat (pharynx or larynx), skin, or kidney/urinary cancer treated with checkpoint inhibitor therapy. Utilizing the Veterans Affairs Informatics and Computing Infrastructure, patients were retrospectively evaluated during the period from October 2015 to June 2021. The primary outcome was overall survival (OS). Patients were followed until death or the end of the study period. There were 2316 patients evaluated, including 34 patients who were exposed to checkpoint inhibitors and fluoxetine. Propensity score weighted Cox proportional hazards demonstrated a better OS in fluoxetine-exposed patients than unexposed (HR: 0.59, 95% CI 0.371–0.936). This cohort study among cancer patients treated with checkpoint inhibitor therapy showed a significant improvement in the OS when fluoxetine was used. Because of this study’s potential for selection bias, randomized trials are needed to assess the efficacy of the association of fluoxetine or another anti-NLRP3 drug to checkpoint inhibitor therapy.

## 1. Introduction

The immune system plays a critical role in defending the body against cancer; however, neoplastic cells’ ability to evade immune surveillance is a hallmark of cancer [1]. A major mechanism of immune evasion by cancer cells involves dysregulated expression of immune checkpoint proteins. Inhibition of immune checkpoint proteins has been hailed as a promising therapeutic strategy for activating therapeutic antitumor immunity [2]. Among the plethora of immune checkpoint proteins, inhibition of programmed cell death 1 receptor (PD-1) or its ligand, programmed cell death receptor ligand 1 (PD-L1) has been extensively studied, and clinical trials have established the efficacy and durability of PD-1/PD-L1 inhibitors [3,4,5,6]. Despite the dramatic clinical responses, the benefit of PD-1/PD-L1 inhibitors is limited to a subset of patients due to primary or acquired resistance [7,8].

The mechanism(s) of resistance and evaluating additional targets or adjuvants to enhance PD-1/L1 therapy effectiveness is a critical research area. Recently, several inflammatory cytokines have been implicated in the pathogenesis of immunotherapy resistance. Inhibiting inflammatory cytokines has been proposed as a potential therapeutic strategy to augment the clinical efficacy of immunotherapeutic agents and to expand the number of patients who could benefit from immune checkpoint-inhibiting strategies [9,10,11,12,13]. Among these inflammatory mediators is the NLR pyrin domain-containing protein 3 (NLRP3) inflammasome, an intracellular protein complex of the innate immune system that senses and responds to an array of exogenous and endogenous danger signals [14]. The recently identified tumor-intrinsic PD-L1/NLRP3 inflammasome signaling pathway suggests that NLRP3 inflammasome inhibition could be a therapeutic target when combined with PD-1/PD-L1 immunotherapy [15,16]. PD-1 blockade leads to CD8+ T cell activation and NLRP3 inflammasome activation, which results in the integration of granulocytic myeloid-derived suppressor cells (PMN-MDSCs) into tumors, and, ultimately, a tumor-permissive environment [15,16]. Therefore, preventing NLRP3 inflammasome activation, pharmacologically, could augment the efficacy of PD-1/PD-L1 immunotherapy and potentially improve clinical responses and outcomes [16,17].

The NLRP3 inflammasome has been implicated in the pathogenesis of an array of inflammatory diseases, and the potential of NLRP3 inhibition as a therapeutic strategy for these diseases is highlighted by the multitude of research and clinical trials [18,19,20,21,22,23]. In addition to the myriad of new therapeutic agents developed to target NLRP3, fluoxetine (FLX), an FDA-approved drug for treating clinical depression, has been recently identified to bind [24] and inhibit NLRP3 [24,25,26]. Of note, several preclinical studies have also highlighted both the independent and synergistic antitumor effects of fluoxetine, suggesting its use in cancer therapy [27,28,29,30]. Given the preclinical research suggesting an enhanced therapeutic effect of PD-1/L1 therapies when combined with NLRP3 inhibition, we test the effect of patients taking FLX, a previously discovered NLRP3 inhibitor, on overall survival among patients treated with PD-1/L1 immunotherapy within the U.S. Department of Veterans Affairs.

## 2. Results

### 2.1. Baseline Characteristics

A total of 2316 patients diagnosed with lung (1598), skin (231), throat (182), liver (106), and urinary (199) cancer and treated with anti-PD-1/L1 therapy (atezolizumab, avelumab, durvalumab, nivolumab, or pembrolizumab) were included in the study. Of these PD-1/L1 treated patients, 34 were exposed to fluoxetine when the checkpoint therapy was initiated. Patients were mostly white (77.68%) and male (97.71%), with a mean age of 68 years old. Most patients (1959–84.6%) had an Eastern Cooperative Oncology Group (ECOG) Performance Status (PS) score of 0 or 1 at baseline. Radiation, chemotherapy, or surgery was identified in 2102 (90.8%) patients (Supplementary Table S1).

To reduce selection bias and minimize differences between the cohorts, we used propensity score weighting to create cohorts with similar baseline characteristics. The gradient-boosted propensity model included age, race, gender, body mass index, depression, Charlson Comorbidity Index, SEER stage, ECOG performance scores at the date of diagnosis, primary cancer site, year and type of checkpoint therapy, and the number of prior treatments (Supplementary Table S2). Standardized differences are generally well below 0.2, indicating covariate balance between the groups. However, depression is poorly matched between cohorts (standardized difference = 0.643).

### 2.2. Fluoxetine and PD-1/PD-L1 Therapy Improves Overall Survival (OS) versus PD-1/PD-L1 Alone

**Figure 1:**
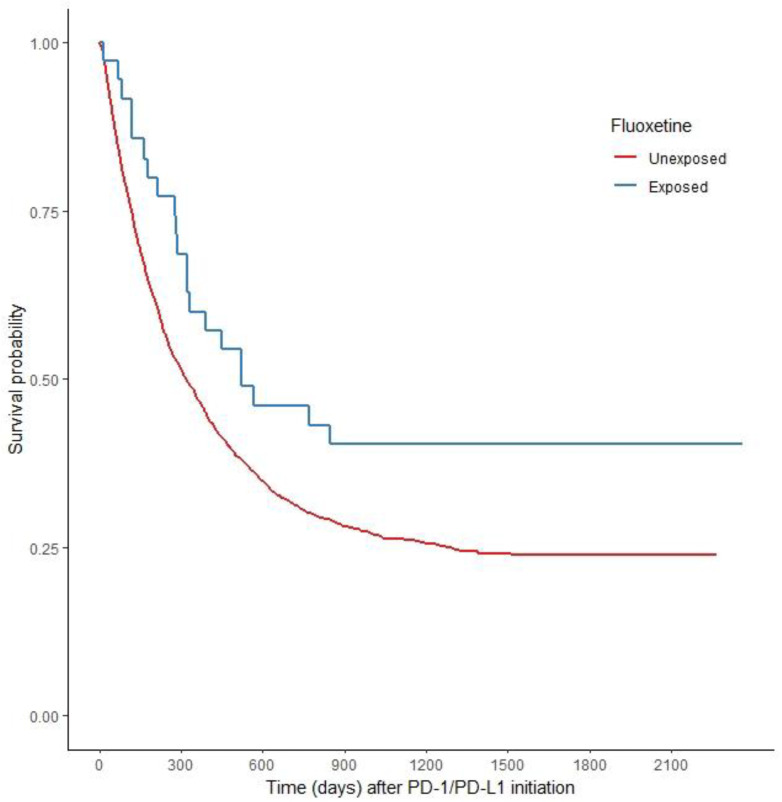
PS weighted Kaplan–Meier Survival Curves.

Figure 1 displays the PS-weighted Kaplan–Meier survival curves. The FLX+PD-1/PD-L1 cohort reveals a consistent survival benefit across the entire follow-up time. The median survival time for those with FLX+PD-1/PD-L1 was 523 days versus 317 days for those with PD-1/PD-L1 alone (Figure 1). Patients exposed to fluoxetine and PD-l/PD-L1 therapy had a significantly better OS than those with PD-1/PD-L1 alone in the PS-weighted Cox proportional hazards model (HR 0.59, 95% CI 0.371–0.936). At the 1-year endpoint, we found a trend toward benefit in OS among the FLX+PD-1/PD-L1 treated patients that did not reach the statistical significance threshold (HR 0.606, 95% CI 0.362–1.015). Patients treated with FLX+PD-1/PD-L1 did have a statistically significant survival advantage, compared to those with a PD-1/PD-L1 alone, at the 2-year endpoint (HR 0.60, 95% CI 0.376–0.958) (Table 1).

As an additional analysis, we present the restricted mean survival time (RMST) calculated on the propensity score matched data. The RMST is a useful measure to quantify the treatment effect without making assumptions about the underlying distribution of survival times. Further, it provides a readily interpretable result in terms of additional survival time. Propensity score matched data were used because RMST analysis is not adjusted for baseline covariates, and matching minimizes the differences between groups allowing a reasonable comparison without multivariate model adjustment. The matched data were generally well balanced, but three covariates did exceed the 0.2 threshold (prior radiation, 0.245; BMI < 18.5, 0.246; depression, 0.635), which is the target for PS matching (Supplementary Table S3). Analysis of the restricted mean survival time (RMST) using the maximum time window (1484 days) showed an average increased survival benefit for those treated with FLX+PD-1/PD-L1 for 287 days (95% CI 48.1–525.8, *p* = 0.019, Table 2). Using a 1-year (365-day) follow-up period, we found an average survival benefit for those treated with FLX+PD-1/PD-L1 of 62.6 days (95% CI 16.6–108.6, *p* = 0.008), and using a 2-year (730-day) follow-up period, we found a survival benefit of 137 days (95% CI 29.7–244.4, *p* = 0.012) (Table 2).

### 2.3. Other Antidepressants Do Not Increase OS

We explored whether two other commonly used antidepressants, sertraline and venlafaxine, had similar OS benefits as fluoxetine when combined with checkpoint therapy. This analysis was conducted to test for a potential generic antidepressant effect and to determine whether there might have been a selection bias among those with an antidepressant medication that could have been a confounder for the beneficial effects observed with fluoxetine use. PS weighted baseline characteristics for sertraline and venlafaxine are displayed in Supplementary Tables S4 and S5. The balance between the cohorts was excellent for the sertraline analysis, with the maximum standardized difference being less than 0.1 (Supplementary Table S4). The balance was not optimal in the venlafaxine analysis, with standardized differences indicating an imbalance in variables such as race, SEER summary, and ECOG performance status (Supplementary Table S5). PS-weighted Cox models are presented in Supplementary Tables S6 and S7. There was no significant improvement in OS with either of these two antidepressants (sertraline, HR 1.069, 95% CI 0.829–1.38, venlafaxine, HR 1.162, 95% CI 0.76–1.776) (Tables S8 and S9). Whether standardized differences were ideal, we adjusted for those variables in our models.

### 2.4. Fluoxetine without PD-1/PD-L1 Therapy Reveals No Benefit on OS

We tested whether fluoxetine without PD-1/PD-L1 therapy could affect OS. We created a new cohort with patients diagnosed with the same cancer types (lung, skin, throat, liver, and urinary) and excluded those treated with any checkpoint therapy. The baseline characteristics of the original sample and the PS-weighted samples are presented in Supplementary Tables S8 and S9, respectively. The balance of the PS-weighted sample was excellent, with the largest standardized difference being 0.175, for the age variable. In the PS-weighted Cox models, we found no statistically significant difference in the OS between patients exposed and unexposed to fluoxetine (HR 1.069, 95% CI 0.933–1.223). The 1-year and 2-year endpoints were consistent with the overall model (1-year HR 1.107 95% CI 0.939–1.305; 2-year HR 1.104 95% CI 0.954–1.276) (Table S10).

## 3. Discussion

PD-1/PD-L1 inhibitors have emerged as a new treatment paradigm for a range of cancer types. These therapies target the PD-1 signaling pathway, including the PD-1 receptor on T cells and the ligand expressed on tumors. PD-1 signaling plays a critical role in modulating the immune response to cancer cells. By blocking this pathway, PD-1/PD-L1 inhibitors enhance the ability of the immune system to recognize and destroy cancer cells. Therapies targeting PD-1 signaling represent a significant advancement in cancer therapy. Despite the remarkable success of PD-1/PD-L1 inhibitors on cancer morbidity and mortality, the benefit of these therapies is limited to a subset of patients because of primary or acquired resistance [7,8]. Thus, there is a critical need to understand the mechanisms of PD-1/PD-L1 resistance and develop strategies to enhance treatment efficacy. The recently identified tumor-intrinsic PD-L1/NLRP3 inflammasome signaling pathway suggests that NLRP3 inflammasome inhibition could be a therapeutic target in combination with PD-1/PD-L1 immunotherapy [15,16].

Preclinical data suggest that myeloid-derived suppressor cells (MDSCs) are responsible for tumor-associated immunosuppression and are highly correlated with poor clinical outcomes [31,32,33,34]. The migration of MDSCs, in particular the migration of granulocytic MDSCs (PMN-MDSCs), to the tumor bed relies on chemokines such as the CXCR2 receptor and blockade of this receptor improved PD-1 efficacy [35,36,37]. Recent reports suggest that the mechanistic link by which MDSCs constrain the immune system response to PD-1/L1 therapy is through the NLRP3/PD-L1 tumor intrinsic signaling pathway [15,16]. A recent study found that in response to PD-1 immune therapy, the tumor intrinsic signaling pathway results in CD8+ T cell activation, which results in the migration of PMN-MDSCs to the tumor bed and, ultimately, resistance to PD-1 therapy [16]. This study also reported that systemic NLRP3 inhibition suppressed PMN-MDSC recruitment and, along with PD-1 therapy, inhibited tumor progression more effectively than PD-1 monotherapy [16].

The NLRP3 inflammasome has been implicated in the pathogenesis of numerous inflammatory diseases, and the potential of NLRP3 inhibition as a therapeutic strategy for these diseases is highlighted by the multitude of clinical trials currently underway [18,19,20,21]. Fluoxetine (FLX), an FDA-approved drug for clinical depression, has been recently identified to possess anti-NLRP3 activity, in contrast to several other antidepressants that do not block NLRP3 [24].

Given these data, we sought to compare the OS between patients who used FLX in combination with PD-1/L1 therapy and those who received PD-1/L1 without FLX. We conducted a retrospective drug–disease study using a national cohort of patients treated with immunotherapy by the U.S. Dept. of Veterans Affairs. Here, we report that, among patients with lung, throat, skin, or kidney/urinary cancer, the combination of fluoxetine and PD-1/L1 therapy is associated with an overall survival benefit compared to PD-1/L1 therapy without fluoxetine. Our findings are consistent with preclinical data showing that NLRP3 inhibitors augment the efficacy of PD-1/L1 inhibitors and suggest that combination therapy could be beneficial for the treatment of these cancers. Our data also suggest the beneficial effects of FLX are limited to when combined with PD-1/L1 immunotherapy as no effect of FLX was found in patients without PD-1/L1 therapy.

While this research reveals a potential survival benefit with FLX and PD-1/L1 treatment, the results should be interpreted cautiously. Importantly, given the limited sample size, we were not able to test for differences in PD-1 and PD-L1 therapy. While both target PD-1 signaling, the two treatments are different in that PD-1 therapies target the PD-1 receptor on T cells, while PD-L1 therapies target the ligand expressed in tumors. The majority of patients in this sample were treated with PD-1 therapies, precluding the testing of differences between the two. Future research could compare NLRP3 inhibition between PD-1 and PD-L1 therapy.

Moreover, while FLX has been shown to directly bind and prevent the activation of NLRP3, this study did not consider other potential NRTI inhibitors, notably nucleoside reverse transcriptase inhibitors (NRTIs). NRTIs are used primarily in the treatment of HIV, and prior studies have shown these medications could have beneficial effects in other disease states. Future research could examine the effects of NRTIs on PD-1/L1 therapy as well. Further, the present study did not consider the effect of FLX dosage. Prior research has shown that the ability of FLX to inhibit NLRP3 is dose-dependent. Moreover, the duration of FLX therapy was not considered in this analysis and could impact overall survival.

Limitations of this study also include those intrinsic to all health insurance claims database analyses. First, medication usage was extracted from filled prescriptions; thus, we cannot verify that patients took these medications as required. Second, the study sample comprised predominately males with a mean age of 68. These findings may not be generalizable to patients with different demographics. Importantly, response to PD-1/L1 therapy could be different between men and women, and future research should consider this possibility. Third, the study utilized data from the Veterans Affairs Informatics and Computing Infrastructure (VINCI); thus, its applicability to other populations is unknown.

Although our study exhibits limitations common to retrospective analysis, our findings are in line with preclinical data supporting the benefit of NLRP3/PD-1 combination therapy. Our study also has many strengths, including the use of patient-level data and the availability of clinical factors from the VA oncology data. In particular, we studied patients in a nationally integrated healthcare system, thereby reducing the potential biases of single-center studies.

As a retrospective study, the possibility of selection bias or residual confounding remains. We performed additional analyses to minimize and evaluate the risk of this bias. We used propensity score weighting, widely used to draw causal inferences in retrospective studies, via a gradient-boosted model, to minimize bias in our results. We further evaluated the possibility of an antidepressant effect or selection bias for those with an antidepressant prescription by extending our analysis to two other NLRP3 non-inhibiting antidepressants, sertraline and venlafaxine. The results of those analyses do not suggest any bias or a greater treatment effect for those with an antidepressant prescription in general. While our findings suggest the potential benefit of combining fluoxetine treatment with cancer immunotherapy, prospective randomized trials are needed to demonstrate the efficacy of FLX+PD-1/L1 treatment compared to monotherapy.

## 4. Materials and Methods

### 4.1. Data Source

We conducted a drug–disease retrospective cohort study examining the overall survival (OS) of cancer patients from the United States Department of Veterans Affairs. We obtained individual-level data on demographics, medical history, and pharmacy dispensation using the Veterans Affairs Informatics and Computing Infrastructure (VINCI). The study was conducted in compliance with the Department of Veterans Affairs requirements and received Institutional Review Board and Research and Development approval.

### 4.2. Cohort Creation

Patients were included in the study if they had a single cancer in the VA oncology data with a primary site of lung, throat (pharynx or larynx), skin, or kidney/urinary and if they had initiated a checkpoint therapy (nivolumab, pembrolizumab, cemiplimab, dostarlimab, atezolizumab, avelumab, durvalumab) prior to 1 January 2020. The study index was based on the first checkpoint therapy prescription, ranging from October 2015 to December 2019. Patients were followed until death or end of study follow-up on 1 June 2021. Patients were enrolled if they were treated with checkpoint therapy as a first or further-line therapy. We excluded patients with (a) multiple tumors in the cancer registry or (b) missing SEER summary status or Eastern Cooperative Oncology Group (ECOG) performance status.

### 4.3. Exposure Definition

Patients were considered fluoxetine-exposed if they had a supply of fluoxetine when initiating PD-1/PD-L1 therapy. Fluoxetine dispenses were extracted from the VA outpatient pharmacy data.

### 4.4. Covariate Data

We included demographic variables such as age, race, sex, year of study entrance, and overall comorbid health burden as measured by the Charlson Comorbidity Index, body mass index (BMI), and depression. We also included clinical information extracted from the VA oncology data related to cancer, including the SEER summary stage, ECOG performance status, cancer type, and prior treatment, including radiation, chemotherapy, and surgery.

### 4.5. Outcomes

The primary outcome was overall survival (OS) and was constructed by evaluating the time to death or study endpoint from PD-1/PD-L1 therapy initiation. The date of death was extracted from the VA vital status files and is a composite of data from sources such as Medicare, the Social Security Administration, and death certificates [38].

### 4.6. Statistical Analysis

To analyze the association between FLX use and overall survival, we first generated summaries of the baseline demographic, comorbid, and clinical characteristics by FLX exposure status. We present means and standard deviations for continuous variables as well as counts and percents for categorical. Categorical variables with cell size counts less than 5 were masked in the summary tables. We utilized *p*-values from the chi-square or *t*-test along with the standardized difference, a more sample size invariant metric, to evaluate the differences among the FLX exposed and unexposed cohorts. The standardized difference was calculated by subtracting the treatment means and then dividing by the pooled standard deviation. We estimated Kaplan–Meier survival curves and adjusted Cox proportional hazards models. Cox models are fit using all follow-up time as well as 1- and 2-year follow-up endpoints.

As a retrospective clinical study, the treatment assignment was not randomized. We utilized inverse probability treatment weights to minimize potential bias from non-random treatment assignments. To estimate the propensity score weights, we used a machine learning algorithm, a generalized boosted model (GBM) implemented in the R package twang [39,40,41]. As an ensemble method, generalized boosted models consist of multiple regression trees, which are aggregated into a final model. We used the standardized mean difference as the model-stopping rule. All covariates were included in the propensity score model. Weighted Kaplan–Meier survival curves and weighted Cox proportional hazards models were estimated. To provide doubly robust estimates, we included all variables included in the propensity score model in the final weighted models.

As an additional analysis, we created propensity score matched cohorts using variable ratio matching, with at least 1 and at most 4 matched controls. We calculated the restricted mean survival time (RMST) using the propensity score matched data. RMST analysis is unadjusted; therefore, we used propensity score matching to minimize the differences between groups. Follow-up times include the overall sample period with a minimum of the greatest survival time as the truncation time, the 1-year endpoint (365-day truncation time), and the 2-year endpoint (730-day truncation time). We present the RSMT, 95% confidence intervals, and mean survival difference between the cohorts, along with associated 95% intervals. We used the R package survRMS2 to estimate the RMST and MatchIt for the matching [42,43].

#### 4.6.1. Other Antidepressants and OS

To account for the possibility of a global antidepressant medication effect, or selection bias for those on antidepressants, we conducted an additional analysis of commonly used antidepressants: sertraline and venlafaxine, which do not inhibit NLRP3 [24]. We replicated the analysis conducted on FLX with both sertraline and venlafaxine. If the results for sertraline or venlafaxine reveal a statistically significant survival benefit, it could be suggestive of an underlying bias in the analysis related to the use of antidepressants among immunotherapy-treated patients.

#### 4.6.2. Secondary Analysis: Fluoxetine without PD-1/PD-L1 Therapy

Further, because NLRP3 inhibition is also found to drive cancer progression independently of checkpoint inhibition, we tested whether patients on FLX without PD-1/PD-L1 were conferred any OS benefit. We extracted a cohort of patients who were each diagnosed with a single cancer in the VA oncology data, with a primary site of lung, throat (pharynx or larynx), skin, or kidney/urinary. Patients who initiated PD-1/PD-L1 therapy were excluded from the analysis. FLX exposure was indicated if patients had a supply of FLX at cancer diagnosis. We compared OS, from cancer diagnosis to study endpoint, in the FLX exposed and unexposed cohorts. The statistical analysis was replicated from the primary analysis. Propensity score weighted samples were generated, and subsequently, weighted Cox models were fit, estimating the hazard ratio for FLX exposure versus no exposure.

## 5. Conclusions

Among patients with lung, throat, skin, or kidney/urinary cancer, combination therapy with fluoxetine, a recently identified direct NLRP3 inhibitor, when taken concurrently with PD-1/L1 therapy, is associated with an overall survival benefit compared to PD-1/L1 therapy alone. These results are consistent with preclinical data suggesting a treatment benefit for the combination of NLRP3 inhibition and PD-1/L1 therapy beyond PD-1/L1 monotherapy. Additional studies and prospective trials are warranted.

## Figures and Tables

**Table 1:** Propensity score weighted Cox model: All cancers.

Variable	All Follow Up HR (95% CI)	1 yr HR (95% CI)	2 yr HR (95% CI)
FLX +PD-1/L1 vs. PD-1/L1 alone	0.59 (0.371–0.936)	0.606 (0.362–1.015)	0.6 (0.376–0.958)
Age	1.002 (0.997–1.008)	1.001 (0.995–1.008)	1.002 (0.997–1.008)
Race: Other/unknown vs. Black	1.569 (1.188–2.072)	1.781 (1.331–2.382)	1.587 (1.195–2.108)
White vs. Black	1.282 (1.128–1.456)	1.33 (1.137–1.555)	1.258 (1.103–1.435)
Sex: Male vs. Female	0.958 (0.703–1.306)	1.056 (0.711–1.571)	0.977 (0.703–1.357)
BMI: 18.5–24.9 vs. <18.5	0.779 (0.648–0.936)	0.85 (0.678–1.066)	0.809 (0.666–0.983)
25–29.9 vs. <18.5	0.655 (0.543–0.79)	0.691 (0.547–0.874)	0.678 (0.554–0.829)
30+ vs. <18.5	0.669 (0.55–0.814)	0.687 (0.538–0.878)	0.684 (0.554–0.844)
Missing vs. <18.5	1.498 (0.498–4.504)	2.377 (0.991–5.704)	1.724 (0.61–4.868)
Depression	0.978 (0.869–1.101)	0.988 (0.86–1.136)	0.989 (0.876–1.117)
Charlson comorbidity index	1.051 (1.036–1.067)	1.06 (1.042–1.08)	1.056 (1.04–1.072)
seer summary: localized vs. distant metastasis	0.978 (0.841–1.137)	0.841 (0.701–1.009)	0.943 (0.804–1.106)
Regional vs. distant metastasis	0.938 (0.837–1.052)	0.863 (0.753–0.989)	0.894 (0.793–1.007)
ECOG performance at diagnosis ECOG 1vs. 0	1.06 (0.953–1.179)	1.088 (0.957–1.238)	1.076 (0.963–1.203)
ECOG 2 vs. 0	1.277 (1.084–1.505)	1.379 (1.14–1.668)	1.281 (1.077–1.523)
ECOG 3 vs. 0	1.544 (1.134–2.102)	1.748 (1.278–2.39)	1.54 (1.118–2.12)
ECOG 4 vs. 0	1.971 (0.784–4.956)	2.081 (0.805–5.383)	2.194 (0.951–5.062)
Liver vs. Kidney/other urinary	1.627 (1.212–2.185)	1.696 (1.205–2.388)	1.704 (1.269–2.287)
Lung vs. Kidney/other urinary	1.631 (1.356–1.961)	1.699 (1.341–2.151)	1.668 (1.37–2.031)
Skin vs. Kidney/other urinary	0.699 (0.538–0.909)	0.861 (0.622–1.191)	0.705 (0.531–0.937)
Throat vs. Kidney/other urinary	1.487 (1.151–1.919)	1.471 (1.067–2.026)	1.529 (1.172–1.994)
Year of PD-1/PD-L1 start	0.85 (0.811–0.892)	0.924 (0.874–0.977)	0.877 (0.834–0.922)
AVELUMAB	0.8 (0.156–4.116)	0.975 (0.209–4.545)	0.882 (0.174–4.464)
DURVALUMAB	0.241 (0.151–0.386)	0.169 (0.092–0.309)	0.252 (0.157–0.406)
NIVOLUMAB	0.811 (0.591–1.113)	0.739 (0.536–1.019)	0.813 (0.59–1.12)
PEMBROLIZUMAB	0.66 (0.476–0.914)	0.588 (0.422–0.819)	0.66 (0.475–0.918)
Total prior TX 2 vs. 1	1.153 (1.028–1.295)	1.122 (0.979–1.286)	1.154 (1.023–1.302)
3 vs. 1	1.091 (0.812–1.465)	1.12 (0.792–1.585)	1.122 (0.824–1.526)
0 vs. 1	0.928 (0.763–1.129)	0.952 (0.755–1.201)	0.938 (0.764–1.15)

**Table 2:** Restricted mean survival time (RMST) in days; Propensity score matched cohort.

	Exposure	Restricted Mean Survival Times (95% CI)	Mean Survival Difference (95% CI)	*p*-Value
All follow-up (1484-day truncation time)	FLX+PD-1/L1	794.6 (593.5–995.8)	287 (48.1–525.8)	0.019
	PD-1/L1	507.7 (378.8–636.5)		
1 year (365-day truncation time)	FLX+PD-1/L1	296.7 (261.5–332)	62.6 (16.6–108.6)	0.008
	PD-1/L1	234.1 (204.6–263.7)		
2 year (730-day truncation time)	FLX+PD-1/L1	482.2 (395.4–569)	137.1 (29.7–244.4)	0.012
	PD-1/L1	345.2 (282.1–408.2)		

## Data Availability

These analyses were performed using data that are available within the US Department of Veterans Affairs secure research environment, the VA Informatics and Computing Infrastructure (VINCI). All relevant data outputs are within the paper and its Supplemental Information.

## Supplementary Materials

The following supporting information can be downloaded at: https://www.mdpi.com/article/10.3390/ph16050640/s1, Table S1: Baseline characteristics. Table S2: PS weighted baseline characteristics. Table S3: Matched sample characteristics. PS weighted baseline characteristics: Sertraline +PD-1/PD-L1 vs. PD-1/L1 alone. Table S4: PS weighted baseline characteristics: Sertraline +PD-1/PD-L1 vs. PD-1/L1 alone. Table S5: PS weighted baseline characteristics: Venlafaxine +PD-1/PD-L1 vs. PD-1/PD-L1 alone. Table S6: PS weighted Cox model: Sertraline +PD-1/L1 vs. PD-1/L1 alone. Table S7: PS weighted Cox model: Venlafaxine +PD-1/L1 vs. PD-1/L1 alone. Table S8: Baseline characteristics among patients without PD-1/L1 therapy. Table S9: PS-weighted baseline characteristics among patients without PD-1/L1 therapy. Table S10: PS-weighted Cox models. FLX and OS among patients without PD-1/L1 therapy.
