# Supplementary material for: Association between Fluoxetine Use and Overall Survival among Patients with Cancer Treated with PD-1/L1 Immunotherapy
Source: Pharmaceuticals (Basel). 2023 Apr 23;16(5):640. doi: 10.3390/ph16050640 (PMC10220705; doi:10.3390/ph16050640)
Supplement: Supplementary file 1 [file pharmaceuticals-16-00640-s001.zip › pharmaceuticals-2313687-supplementary.pdf]

Supplementary Information for

Association between Fluoxetine Use and Overall Survival among Patients with Cancer Treated with PD-1/L1 Immunotherapy

Joseph Magagnoli [1,2], Siddharth Narendran [3,4], Felipe Pereira [3,4], Tammy H. Cummings [1,2], James W. Hardin [1,5], S. Scott Sutton [1,2,*] and Jayakrishna Ambati [3,4,6,7,*]

[1] Dorn Research Institute, Columbia VA Health Care System, Columbia, SC 29209, USA; magagnol@mailbox.sc.edu (J.M.); harris68@mailbox.sc.edu (T.H.C.); jhardin@mailbox.sc.edu (J.W.H.)
[2] Department of Clinical Pharmacy and Outcomes Sciences, College of Pharmacy, University of South Carolina, Columbia, SC,29208, USA
[3] Center for Advanced Vision Science, University of Virginia School of Medicine, Charlottesville, VA 22903, USA; siddhu_12345@yahoo.com (S.N.); felipe.oft@gmail.com (F.P.)
[4] Department of Ophthalmology, University of Virginia School of Medicine, Charlottesville, VA 22903, USA
[5] Department of Epidemiology & Biostatistics, University of South Carolina, Columbia, SC 29208, USA
[6] Department of Pathology, University of Virginia School of Medicine, Charlottesville, VA 22903, USA
[7] Department of Microbiology, Immunology, and Cancer Biology, University of Virginia School of Medicine, Charlottesville, VA 22903, USA
* Correspondence: ja9qr@virginia.edu (J.A.); sutton@cop.sc.edu (S.S.S.)

Supplementary Table S1. Baseline characteristics.

	Variable	PD-1/L1 alone N=2282	FLX +PD-1/PD-L1 exposed N=34	P value	Standardized difference
Age		68.07(9.23)	68.65(11.24)	0.749	0.062
Race	Black	423(18.54%)	5(14.71%)	0.603	0.099
	Other/unknown	87(3.81%)	#(<10%)		0.108
	White	1772(77.65%)	27(79.41%)		0.042
Sex	Female	53(2.32%)	0(0%)	1	0.155
	Male	2229(97.68%)	34(100%)	1	0.155
BMI	<18.5	159(6.97%)	#(<10%)	0.877	0.159
	18.5-24.9	844(36.99%)	14(41.18%)		0.087
	25-29.9	722(31.64%)	10(29.41%)		0.048
	30+	547(23.97%)	9(26.47%)		0.059
	Missing	10(0.44%)	0(0%)		0.067
Depression		544(23.84%)	18(52.94%)	<0.001	0.679
Charlson		8.45(3.28)	8.56(2.78)	0.402	0.034
SEER summary stage	Distant metastasis	982(43.03%)	15(44.12%)	0.816	0.022
	Localized	392(17.18%)	7(20.59%)		0.09
	Regional	908(39.79%)	12(35.29%)		0.092
ECOG performance at diagnosis	ECOG 0	924(40.49%)	13(38.24%)	0.99	0.046
	ECOG 1	1006(44.08%)	16(47.06%)		0.06
	ECOG 2	270(11.83%)	#(<12%)		0.002
	ECOG 3	75(3.29%)	#(<10%%)		0.019
	ECOG 4	7(0.31%)	0(0%)		0.056
Cancer type	Kidney & other urinary	196(8.59%)	#(<10%)	0.571	0.008
	LIVER	103(4.51%)	#(<10%)		0.206
	Lung	1575(69.02%)	23(67.65%)		0.03
	Skin	227(9.95%)	#(<12%)		0.061
	Throat	181(7.93%)	#(<10%)		0.185

Year of PD-1/PD-L1 start			2017.07(1.16)	2017.03(1.09)	0.983	0.039
Prior radiation			1016(44.52%)	11(32.35%)	0.214	0.245
Prior chemotherapy			1503(65.86%)	24(70.59%)	0.693	0.1
Prior surgery			612(26.82%)	11(32.35%)	0.598	0.125
Total prior treatment		1	1069(46.84%)	16(47.06%)	1	0.004
		2	944(41.37%)	15(44.12%)		0.056
		3	58(2.54%)	0(0%)		0.163
		None	211(9.25%)	#(<10%)		0.015
PD-1/PD-L1	Atezolizumab		64(2.8%)	#(<10%)	0.68	0.008
	Avelumab		5(0.22%)	0(0%)		0.047
	Durvalumab		77(3.37%)	#(<10%)		0.138
	Nivolumab		1472(64.5%)	21(61.76%)		0.057
	Pembrolizumab		664(29.1%)	10(29.41%)		0.007

# indicates masked cell sample

Supplementary Table S2. PS weighted baseline characteristics.

Variable		PD-1/L1 alone	FLX +PD-1/L1 exposed	Standardized difference
Age		68.074	68.576	0.054
Race	Black	19%	15%	0.105
	Other/unknown	4%	6%	0.087
	White	78%	80%	0.058
Sex	Female	2%	0%	0.155
	Male	98%	100%	0.155
BMI	<18.5	7%	3%	0.166
	18.5-24.9	37%	40%	0.057
	25-29.9	32%	32%	0.001
	30+	24%	26%	0.046
	Missing	0%	0%	0.067
Depression		24%	52%	0.643
Charlson		8.447	8.577	0.04
SEER summary stage	Distant metastasis	43%	46%	0.052
	Localized	17%	20%	0.072
	Regional	40%	35%	0.109
ECOG performance at diagnosis	ECOG 0	41%	37%	0.066
	ECOG 1	44%	49%	0.091
	ECOG 2	12%	11%	0.016
	ECOG 3	3%	3%	0.025
	ECOG 4	0%	0%	0.056
Cancer type	Kidney & other urinary	9%	9%	0.001
	Liver	5%	8%	0.183
	Lung	69%	69%	0.005
	Skin	10%	11%	0.038
	Throat	8%	3%	0.193
Year of PD-1/PD-L1 start		2017.074	2016.996	0.067
PD-1/PD-L1	Atezolizumab	3%	3%	0.006
	Avelumab	0%	0%	0.047
	Durvalumab	3%	6%	0.117
	Nivolumab	65%	64%	0.015
	Pembrolizumab	29%	28%	0.024
Total prior treatment (radiation/chemo/surg)	1	47%	48%	0.02
	2	41%	44%	0.049
	3	3%	0%	0.163
	None	9%	8%	0.03

Supplementary Table 3. Matched sample characteristics.

	Variable	PD-1/L1 alone N=68	FLX+PD-1/L1 N=34	P.value	Standardized difference
Age		68.32(6.34)	68.65(11.24)	0.639	0.035
Race	Black	6(8.82%)	5(14.71%)	0.699	0.183
	Other/Unknown	#(#)	#(#)	0.699	0
	White	58(85.29%)	27(79.41%)	0.699	0.155
Sex	Female	0(0%)	0(0%)	1	0
	Male	68(100%)	34(100%)	1	0
BMI	<18.5	0(0%)	#(#)	0.675	0.246
	18.5-24.9	30(44.12%)	14(41.18%)	0.675	0.059
	25-29.9	19(27.94%)	10(29.41%)	0.675	0.033
	30+	19(27.94%)	9(26.47%)	0.675	0.033
	Missing	0(0%)	0(0%)	0.675	0
Depression		16(23.53%)	18(52.94%)	0.006	0.635
Charlson		8.53(3.41)	8.56(2.78)	0.583	0.009
Seer summary stage	Distant metastasis	36(52.94%)	15(44.12%)	0.563	0.177
	Localized	9(13.24%)	7(20.59%)	0.563	0.197
	Regional	23(33.82%)	12(35.29%)	0.563	0.031
ECOG performance at diagnosis	ECOG 0	21(30.88%)	13(38.24%)	0.859	0.155
	ECOG 1	32(47.06%)	16(47.06%)	0.859	0
	ECOG 2	12(17.65%)	#(#)	0.859	0.167
	ECOG 3	#(#)	#(#)	0.859	0.078
	ECOG 4	0(0%)	0(0%)	0.859	0
Cancer type	Kidney & Other Urinary	5(7.35%)	#(#)	1	0.054
	Liver	8(11.76%)	#(#)	1	0.097
	Lung	46(67.65%)	23(67.65%)	1	0
	Skin	7(10.29%)	#(#)	1	0.047
	Throat	#(#)	#(#)	1	0
Year of PD-1/PD-L1 start		2017.01(1.07)	2017.03(1.09)	0.767	0
Prior radiation		30(44.12%)	11(32.35%)	0.353	0.244
Prior chemotherapy		51(75%)	24(70.59%)	0.812	0.099
Prior surgery		14(20.59%)	11(32.35%)	0.29	0.269
Total prior treatment (radiation/chemo/surg)	1	27(39.71%)	16(47.06%)	0.826	0.149
	2	34(50%)	15(44.12%)	0.826	0.118
	3	0(0%)	0(0%)	0.826	0
	None	7(10.29%)	#(#)	0.826	0.05
PD-1/PD-L1	Atezolizumab	#(#)	#(#)	0.943	0
	Avelumab	0(0%)	0(0%)	0.943	0
	Durvalumab	#(#)	#(#)	0.943	0
	Nivolumab	46(67.65%)	21(61.76%)	0.943	0.123
	Pembrolizumab	16(23.53%)	10(29.41%)	0.943	0.134

# indicates masked cell sample

Supplementary Table S4. PS weighted baseline characteristics: Sertraline +PD-1/PD-L1 vs. PD-1/L1 alone

Variable		PD-1/L1 alone	Sertraline +PD-1/L1	Standardized difference
Age		68.109	67.422	0.074
Race	Black	19%	18%	0.027
	Other/unknown	4%	5%	0.062
	White	78%	77%	0.004
Sex	Female	2%	2%	0.045
	Male	98%	98%	0.045
BMI	<18.5	7%	7%	0.017
	18.5-24.9	37%	39%	0.041
	25-29.9	31%	32%	0.012
	30+	24%	22%	0.038
	Missing	0%	0%	0.069
Depression		24%	27%	0.06
Charlson		8.447	8.374	0.022
Seer summary stage	Distant	43%	42%	0.036
	Localized	17%	19%	0.053
	Regional	40%	39%	0.004
ECOG performance at	ECOG 0	41%	42%	0.024
	ECOG 1	44%	43%	0.016
	ECOG 2	12%	12%	0.01
	ECOG 3	3%	3%	0.022
	ECOG 4	0%	0%	0.058
Cancer type	Kidney & other	9%	11%	0.069
	Liver	5%	5%	0.013
	Lung	69%	69%	0
	Skin	10%	9%	0.027
	Throat	8%	7%	0.052
Year of PD-1/PD-L1 start		2017.072	2017.157	0.074
Total prior treatment	1	47%	45%	0.045
	2	42%	45%	0.064
	3	3%	2%	0.06
	None	9%	9%	0.001
PD-1/PD-L1	Atezolizumab	3%	3%	0.028
	Avelumab	0%	0%	0.048
	Durvalumab	4%	3%	0.047
	Nivolumab	64%	65%	0.013
	Pembrolizumab	29%	29%	0.001

Supplementary Table S5. PS weighted baseline characteristics: Venlafaxine +PD-1/PD-L1 vs. PD-1/PD-L1 alone

		PD-1/L1 alone	Venlafaxine + PD-1/L1	Standardized difference
Age		68.069	69.289	0.132
Race	Black	19%	4%	0.375
	Other/Unknown	4%	1%	0.15
	White	78%	95%	0.419
Sex	Female	2%	2%	0.003
	Male	98%	98%	0.003
BMI	<18.5	7%	0%	0.275
	18.5-24.9	37%	40%	0.056
	25-29.9	32%	26%	0.12
	30+	24%	34%	0.24
	Missing	0%	0%	0.066
Depression		24%	25%	0.01
Charlson		8.447	9.68	0.377
SEER summary stage	Distant metastasis	43%	29%	0.282
	Localized	17%	26%	0.244
	Regional	40%	44%	0.097
ECOG performance at	ECOG 0	40%	53%	0.259
	ECOG 1	44%	30%	0.28
	ECOG 2	12%	9%	0.1
	ECOG 3	3%	8%	0.264
	ECOG 4	0%	0%	0.056
Cancer type	Kidney/other urinary	2017.069	2017.23	0.139
	Liver	9%	11%	0.08
	Lung	5%	0%	0.221
	Skin	69%	71%	0.039
	Throat	10%	17%	0.234
Year of PD-1/PD-L1 start		8%	1%	0.24
Total prior treatment	1	47%	60%	0.265
	2	41%	34%	0.153
	3	3%	0%	0.163
	None	9%	6%	0.108
PD-1/PD-L1	Atezolizumab	3%	1%	0.104
	Avelumab	0%	0%	0.047
	Durvalumab	3%	1%	0.133
	Nivolumab	65%	73%	0.173
	Pembrolizumab	29%	25%	0.087

Supplementary Table S6. PS weighted Cox model: Sertraline +PD-1/L1 vs. PD-1/L1 alone

Variable	All follow up HR(95% CI)
Sertraline +PD-1/L1 vs. PD-1/L1 alone	1.069 (0.829-1.38)
Age	1.007 (0.999-1.014)
Race: Other/unknown vs. Black	2.618 (1.642-4.174)
White vs. Black	1.41 (1.11-1.793)
Sex: Male vs. Female	0.9 (0.597-1.355)
BMI: 18.5-24.9 vs <18.5	0.774 (0.584-1.026)
25-29.9 vs. <18.5	0.746 (0.567-0.981)
30+ vs. <18.5	0.737 (0.543-1.001)
Missing vs. <18.5	1.486 (0.435-5.079)
Depression	1.033 (0.858-1.244)
Charlson	1.055 (1.028-1.081)
SEER summary: Localized vs distant metastasis	0.692 (0.521-0.92)
Regional vs distant metastasis	0.883 (0.701-1.113)
ECOG performance at diagnosis ECOG 1 vs. 0	1.024 (0.833-1.259)
ECOG 2 vs. 0	1.434 (1.123-1.831)
ECOG 3 vs. 0	1.787 (1.282-2.491)
ECOG 4 vs. 0	1.885 (0.71-5.005)
Liver vs Kidney/other urinary	1.782 (0.974-3.261)
Lung vs Kidney/other urinary	2.014 (1.268-3.198)
Skin vs Kidney/other urinary	0.888 (0.469-1.683)
Throat vs Kidney/other urinary	2.542 (1.442-4.483)
Year of PD-1/PD-L1 start	0.894 (0.82-0.975)
Total Prior Tx 2 vs. 1	0.917 (0.743-1.133)
3 vs. 1	1.069 (0.711-1.607)
0 vs. 1	0.668 (0.487-0.917)
Avelumab	1.502 (0.258-8.757)
Durvalumab	0.798 (0.276-2.307)
Nivolumab	1.322 (0.71-2.462)
Pembrolizumab	1.12 (0.588-2.134)

Supplementary Table S7. PS weighted Cox model: Venlafaxine +PD-1/L1 vs. PD-1/L1 alone

Variable	All follow up HR(95% CI)
Venlafaxine	1.162 (0.76-1.776)
Age	0.982 (0.964-1)
Race: Other/unknown vs. Black	1.665 (1.098-2.525)
White vs. Black	1.506 (1.222-1.856)
Sex: Male vs. Female	2.389 (0.791-7.211)
BMI: 18.5-24.9 vs <18.5	0.868 (0.68-1.108)
25-29.9 vs. <18.5	0.526 (0.382-0.722)
30+ vs. <18.5	0.602 (0.439-0.825)
Missing vs. <18.5	1.452 (0.437-4.829)
Depression	0.786 (0.614-1.007)
Charlson	1.066 (1.04-1.092)
SEER summary: Localized vs distant metastasis	1.024 (0.663-1.58)
Regional vs distant metastasis	1.242 (1.008-1.531)
ECOG performance at diagnosis: ECOG 1 vs. 0	1.178 (0.935-1.484)
ECOG 2 vs. 0	1.441 (1.066-1.948)
ECOG 3 vs. 0	0.368 (0.113-1.198)
ECOG 4 vs. 0	2.014 (0.741-5.471)
Liver vs Kidney/other urinary	3.86 (1.357-10.983)
Lung vs Kidney/other urinary	5.016 (1.707-14.742)
Skin vs Kidney/other urinary	1.696 (0.551-5.221)
Throat vs Kidney/other urinary	3.374 (1.169-9.738)
Year of PD-1/PD-L1 start	0.868 (0.772-0.975)
Total Prior Tx 2 vs. 1	0.959 (0.796-1.155)
3 vs. 1	0.857 (0.587-1.253)
0 vs. 1	0.884 (0.618-1.263)
Avelumab	0.688 (0.106-4.453)
Durvalumab	0.14 (0.075-0.263)
Nivolumab	0.708 (0.442-1.132)
Pembrolizumab	0.381 (0.229-0.633)

Supplementary Table S8. Baseline characteristics among patients without PD-1/L1 therapy

	Variable	FLX unexposed N=42699	FLX exposed N=828	P-value	Standardized difference
Age		67.29(10.45)	65.46(10.18)	<0.001	0.175
Race	BLACK	6322(14.81%)	84(10.14%)	<0.001	0.132
	Other/unknown	3105(7.27%)	69(8.33%)		0.041
	White	33272(77.92%)	675(81.52%)		0.087
Sex	Female	833(1.95%)	37(4.47%)	<0.001	0.18
	Male	41866(98.05%)	791(95.53%)		0.18
BMI	<18.5	2223(5.21%)	24(2.9%)	<0.001	0.104
	18.5-24.9	14255(33.38%)	216(26.09%)		0.155
	25-29.9	13762(32.23%)	277(33.45%)		0.026
	30+	12041(28.2%)	308(37.2%)		0.2
	Missing	418(0.98%)	#(<10%)		0.063
Depression		12339(28.9%)	574(69.32%)	<0.001	0.885
Charlson		2.74(2.57)	2.96(2.52)	0.001	0.083
Seer summary	Distant	13518(31.66%)	224(27.05%)	0.016	0.099
	Localized	16516(38.68%)	348(42.03%)		0.069
	Regional	12665(29.66%)	256(30.92%)		0.028
ECOG	ECOG 0	16987(39.78%)	349(42.15%)	0.05	0.048
	ECOG 1	15560(36.44%)	285(34.42%)		0.042
	ECOG 2	5923(13.87%)	132(15.94%)		0.06
	ECOG 3	3456(8.09%)	52(6.28%)		0.067
	ECOG 4	773(1.81%)	10(1.21%)		0.045
Cancer type	Kidney & other	4216(9.87%)	110(13.29%)	0.015	0.114
	LIVER	4957(11.61%)	87(10.51%)		0.034
	Lung	22766(53.32%)	439(53.02%)		0.006
	Skin	5055(11.84%)	96(11.59%)		0.008
	Throat	5705(13.36%)	96(11.59%)		0.052
Year of Diagnosis		2012.55(2.95)	2012.85(2.9)	0.006	0.101

Supplementary Table S9. PS-weighted baseline characteristics among patients without PD-1/L1 therapy

		FLX unexposed	FLX exposed	Standardized difference
Age		67.256	67.16	0.009
Race	Black	15%	10%	0.13
	Other/Unknown	7%	8%	0.027
	White	78%	82%	0.094
Sex	Female	2%	2%	0.005
	Male	98%	98%	0.005
BMI	<18.5	5%	3%	0.092
	18.5-24.9	33%	33%	0.014
	25-29.9	32%	34%	0.045
	30+	28%	30%	0.032
	Missing	1%	0%	0.088
Depression		30%	31%	0.031
Charlson		2.748	2.74	0.003
Seer summary stage	Distant metastasis	32%	29%	0.049
	Localized	39%	40%	0.025
	Regional	30%	31%	0.023
ECOG performance at	ECOG 0	40%	38%	0.032
	ECOG 1	36%	40%	0.064
	ECOG 2	14%	14%	0.001
	ECOG 3	8%	7%	0.051
	ECOG 4	2%	2%	0.007
Cancer type	Kidney & other	10%	9%	0.021
	LIVER	12%	9%	0.072
	Lung	53%	57%	0.073
	Skin	12%	12%	0.008
	Throat	13%	13%	0.012
Year of Diagnosis		2012.557	2012.542	0.005

Supplementary Table S10. PS-weighted Cox models. FLX and OS among patients without PD-1/L1 therapy

Variable	All follow-up HR(95% CI)	1 year HR(95% CI)	2 year HR(95% CI)
Fluoxetine	1.069 (0.933-1.223)	1.107 (0.939-1.305)	1.104 (0.954-1.276)
Age	1.016 (1.011-1.021)	1.014 (1.007-1.021)	1.013 (1.007-1.019)
Race: Other/unknown vs. Black	1.063 (0.868-1.303)	1.098 (0.816-1.476)	1.128 (0.89-1.429)
WHITE vs. Black	1.067 (0.938-1.213)	1.163 (0.947-1.429)	1.147 (0.967-1.362)
sex: Male vs. Female	1.399 (1.044-1.876)	1.372 (0.864-2.18)	1.533 (0.962-2.443)
BMI: 18.5-24.9 vs <18.5	0.794 (0.635-0.992)	0.812 (0.623-1.058)	0.795 (0.627-1.008)
25-29.9 vs. <18.5	0.739 (0.597-0.916)	0.732 (0.559-0.957)	0.755 (0.598-0.952)
30+ vs. <18.5	0.659 (0.528-0.822)	0.66 (0.5-0.872)	0.638 (0.5-0.815)
Missing vs. <18.5	1.067 (0.755-1.508)	1.234 (0.895-1.702)	1.122 (0.786-1.601)
Depression	1.004 (0.911-1.107)	0.968 (0.857-1.092)	1 (0.9-1.112)
Charlson	1.035 (1.013-1.058)	1.033 (1.009-1.058)	1.034 (1.012-1.056)
Seer summary: Localized vs distant metastasis	0.214 (0.179-0.257)	0.172 (0.134-0.219)	0.177 (0.145-0.216)
Regional vs distant metastasis	0.433 (0.371-0.505)	0.387 (0.326-0.46)	0.409 (0.352-0.477)
ECOG performance at diagnosis ECOG 1vs. 0	1.492 (1.308-1.701)	1.618 (1.326-1.973)	1.571 (1.342-1.839)
ECOG 2 vs. 0	2.354 (2.035-2.722)	2.638 (2.144-3.247)	2.569 (2.178-3.029)
ECOG 3 vs. 0	3.33 (2.745-4.038)	3.936 (3.061-5.06)	3.73 (3.035-4.585)
ECOG 4 vs. 0	6.046 (4.532-8.066)	6.714 (4.865-9.264)	6.451 (4.793-8.683)
LIVER vs Kidney/other urinary	3.084 (2.544-3.74)	3.498 (2.583-4.738)	3.136 (2.537-3.875)
Lung s Kidney/other urinary	1.93 (1.627-2.29)	2.191 (1.652-2.906)	1.953 (1.624-2.348)
SKIN vs Kidney/other urinary	0.817 (0.655-1.019)	0.763 (0.487-1.196)	0.766 (0.564-1.04)
Throat vs Kidney/other urinary	0.989 (0.802-1.219)	0.989 (0.692-1.415)	1.004 (0.782-1.289)
Year of cancer diagnosis	0.94 (0.923-0.957)	0.975 (0.952-1)	0.964 (0.944-0.985)
